# Effectiveness of Risk-Adapted Upper Gastrointestinal Cancer Screening in China: Prospective Cohort Study

**DOI:** 10.2196/62864

**Published:** 2024-10-10

**Authors:** Youqing Wang, Juan Zhu, Huizhang Li, Le Wang, Chen Zhu, Xue Li, Shi Wang, Lingbin Du

**Affiliations:** 1 Department of Cancer Prevention, Zhejiang Cancer Hospital, Hangzhou Institute of Medicine (HIM), Chinese Academy of Sciences, Hangzhou, 310022, China, 86 13588114180; 2 Department of Endoscopy, Zhejiang Cancer Hospital, Hangzhou Institute of Medicine (HIM), Chinese Academy of Sciences, Hangzhou, China

**Keywords:** upper gastrointestinal cancer, screening, endoscopy, effectiveness, incidence, mortality, all-cause mortality

## Abstract

**Background:**

Previous studies have proved the effectiveness of endoscopic screening in rural areas; however, long-term, high-quality evidence regarding the effectiveness of risk-adapted upper gastrointestinal cancer (UGC) sequential screening strategies in resource-rich regions is currently lacking.

**Objective:**

The objectives were to validate the effectiveness of risk-adapted sequential screening strategies in UGC prevention and control and assess the potential of sequential screening to lower mortality rates.

**Methods:**

Based on the Cancer Screening Program in Urban China, a prospective, large-scale cohort study based on population was conducted to recruit individuals from 4 cities in China from 2013‐2019. Those identified as having a high risk of UGC according to a validated risk-score model were advised to undergo endoscopy tests. Follow-up outcomes were tracked until June 2021. Incidence of UGC, UGC-related mortality, and all-cause mortality were evaluated between the screened and nonscreened cohorts.

**Results:**

The study included 153,079 participants at baseline. In total, 113,916 (74.42%) of the participants were designated as low risk of UGC. The remaining 39,163 (25.68%) participants were deemed to be at high risk of UGC and were offered gastroscopy tests. Among the high-risk participants, 9627 (compliance rate 24.6%) adhered to the gastroscopy tests. Over a median follow-up of 6.05 (IQR 3.06‐7.06) years, 622 UGC cases, 180 UGC deaths, and 1958 all-cause death cases were traced. The screened cohort exhibited the highest cumulative incidence of UGC (119.2 per 100,000 person-years), followed by the nonscreened and low-risk cohorts. Obvious reductions in both all-cause mortality and UGC mortality were observed between those who undertook screening (153.7 and 4.7 per 100,000 person-years, respectively) and the nonscreened group (245.3 and 27 per 100,000 person-years, respectively). The screening population showed a significant 36% and 82% reduction in both all-cause mortality (hazard ratio [HR] 0.64, 95% CI 0.49‐0.83, *P*<.001) and UGC mortality (HR 0.18, 95% CI 0.04‐0.74, *P*=.02), respectively, compared to the nonscreened group. Reductions of 35% in all-cause mortality (HR 0.65, 95% CI 0.49‐0.86, *P*=.003) and 81% in UGC mortality (HR 0.19, 95% CI 0.05‐0.80, *P*=.02) were observed in participants aged older than 55 years in the screened group compared to the nonscreened group. The reductions in all-cause mortality and UGC mortality were statistically significant in males (all-cause mortality: HR 0.64, 95% CI 0.47‐0.88, *P*=.005; UGC mortality: HR 0.10, 95% CI 0.01‐0.72, *P*=.02), but significant reductions were not observed in females (all *P* values were >.05).

**Conclusions:**

Our study suggests the significance of one-off risk-adapted UGC screening in reducing both all-cause mortality and UGC mortality, particularly among high-risk individuals, indicating its effectiveness in UGC prevention and management.

## Introduction

Upper gastrointestinal cancer (UGC), encompassing esophageal cancer (EC) and gastric cancer (GC), represents a significant global public health concern. As per GLOBOCAN 2022 data, EC stands as the seventh most common cause of cancer-related deaths worldwide, whereas GC holds the fifth position in both incidence and mortality worldwide [1]. With substantial efforts to combat UGC in recent years, both incidence and mortality rates have shown noteworthy declines in China [2]. However, UGC continues to impose a substantial burden, persistently ranking within the top 4 for cancer-attributable disability-adjusted life years [3,4]. Thus, UGC remains a formidable challenge for both health care systems and individuals.

Patients diagnosed with late-stage UGC still have a grim prognosis, characterized by a low resection rate and unfavorable survival outcomes [5]. Conversely, for early-stage UGC, a promising prognosis exists through complete surgical removal, resulting in 5-year survival rates exceeding 85% [6,7]. Prior studies have emphasized the substantial potential of gastroscopic screening in alleviating the UGC burden [8-12]. Organized and opportunistic screening programs have been extensively adopted in Asian nations characterized by a high burden of UGC, such as Japan, Korea, and China [8,13-15]. Nonetheless, gastroscopy examination remains an invasive and expertise-intensive procedure, limiting its utility in regions with lower UGC incidence and constrained health care resources. For such regions, the adoption of a risk-stratified scoring system is advised for the identification of high-risk individuals who would benefit from endoscopic evaluation [16,17].

Initiated by the Chinese government in October 2012, the Cancer Screening Program in Urban China (CanSPUC) targets the predominant cancers, including UGC [18,19]. Since 2013, Zhejiang Province has participated in the CanSPUC. This program seeks to provide free cancer screening to eligible participants and uses a cancer risk stratification system to evaluate cancer risk. This system guides individuals with a high risk of EC or GC to undergo gastroscopic evaluation at designated hospitals.

Previous studies have proved the effectiveness of endoscopic screening in rural areas [8,9,20]. Nevertheless, evidence regarding the effectiveness of sequential screening strategies in resource-rich regions is currently lacking. Considering China’s large population base and the significant burden on endoscopy resources, conducting large-scale 1-time endoscopic primary screening is not feasible. Guidelines recommend conducting high-risk assessments first to discern screeners with high risk for subsequent endoscopic screening in non–high-incidence areas. However, there exists an absence of long-term, high-quality evidence supporting this approach. Therefore, our study aims to bridge this knowledge gap by analyzing UGC screening data from 2013 to 2019, involving the comparison of 3 distinct groups: low-risk cohort, high-risk but unscreened cohort, and high-risk population that underwent screening. Our primary objectives were to explore the effectiveness of sequential screening in UGC control and assess its potential to lower mortality rates.

## Methods

### Study Design and Participants

We conducted a screening cohort study within the framework of the CanSPUC program. We used the household registration system in local communities to identify eligible permanent residents who were aged 40‐74 years and asymptomatic for UGC with no history of cancer diagnosis. People who were unable to give informed consent, had a prior cancer diagnosis, or were receiving therapy for other serious medical conditions were ineligible for participation. Television broadcasts, brochures, and websites were used to publicize cancer screening programs. Community-based telephone calls or home visits were carried out by trained staff to reach and inform the maximum number of eligible residents.

**Figure 1.**
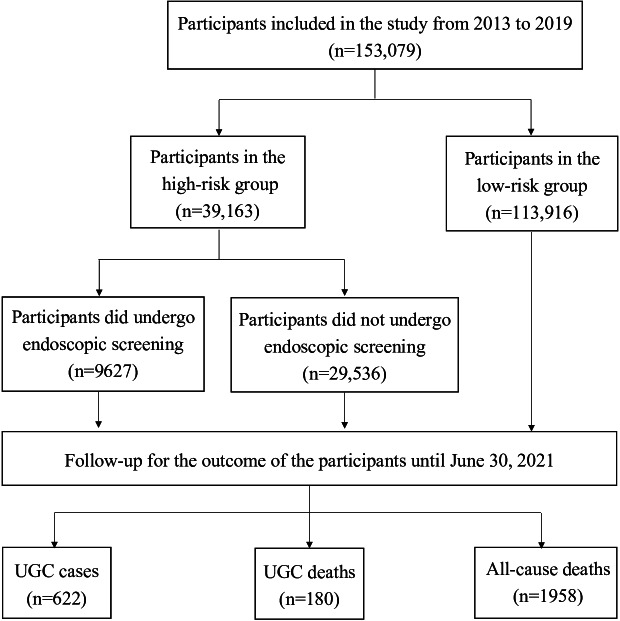
Flow diagram of the cohort study. The study included 153,079 participants at baseline. In total, 74.42% (n=113,916) were evaluated as low risk for UGC, while 25.68% (n=39,163) were deemed to be at high risk and invited for endoscopy tests. Among the high-risk participants, 9627 (compliance rate 24.6%) adhered to the gastroscopy tests. Over a median follow-up of 6.05 (IQR 3.06‐7.06) years, 622 UGC cases, 180 UGC deaths, and 1958 all-cause death cases were traced. UGC: upper gastrointestinal cancer.

We used data from the UGC screening performed over the initial 6 years, spanning from October 2013 to August 2019, in 4 cities within Zhejiang Province: Hangzhou, Ningbo, Quzhou, and Jinhua city. In total, 153,079 eligible participants underwent risk assessment in our UGC screening program. Figure 1 displays a flowchart of the study population.

### Screening Procedure

Participants deemed eligible were initially required to complete a risk evaluation before undergoing endoscopy procedure. To distinguish individuals with a high risk of developing UGC, we used a cancer-related risk assessment questionnaire developed by the CanSPUC group. This questionnaire covered a range of information, including basic information, dietary habits, living environment, lifestyle and habits, psychological and emotional aspects, medical history, and family history of cancer. To evaluate UGC risk, we used gender-specific risk scoring systems derived from the Harvard Cancer Risk Index [21]. A panel of experts assigned a score to each risk factor, considering the strength of its correlation with UGC. These individual risk scores were then totaled and divided by the average risk score in the general population, resulting in a relative risk assessment for each participant. The estimation process is illustrated in detail in Multimedia Appendix 1.

Individuals identified as high risk for UGC were advised to undertake a one-off endoscopy at a specialized tertiary-level hospital designated by the program. Further diagnostic or follow-up procedures were decided depending on the endoscopy results. Any anomalies discovered during the endoscopy were meticulously examined using biopsies. Additionally, visual documentation was made of any endoscopy findings. Individuals who did not have a complete endoscopy examination were encouraged to undergo a repeat endoscopy to fulfill the necessary clinical standards for diagnosis.

### Follow-Up Data

To address potential bias stemming from differences in the time during which participants in the screened cohort were required to stay alive and free from certain events (immortal time), we determined the entry date as the day of screening for participants in the screened group. In the nonscreened group, the cohort entry date was approximated based on the screening date of the participant in the screened group whose risk assessment date most closely matched that of the nonscreened group. The duration until the occurrence of UGC was computed as the time from the entry date to the earliest of 3 events: the development of UGC, death, or administrative censoring (up to June 30, 2021). Similarly, the duration until UGC-specific or all-cause death was calculated as the time from the cohort entry date to the earliest death or administrative censoring.

### Outcome Assessment and Quality Control

Our focus was on 3 key outcomes: new cases of UGC, UGC-related deaths, and deaths from all causes. We identified UGC using the *International Classification of Diseases, 10th Revision*, where EC was coded as C15 and GC as C16. Every 6 months, we collected outcome data from national sources, which included the cancer registry and death surveillance system.

Our data collection involved standardized web-based forms, including an epidemiological questionnaire and endoscopy reports, which were collected by our qualified staff and physicians face-to-face. A thorough verification of consistency was carried out, and any discrepancies were rectified by referring to the original documents. Each participant was assigned a unique identification number, which helped us keep track of all relevant documentation.

### Statistical Analysis

We compared the screened versus nonscreened groups and the high-risk versus low-risk groups using standardized differences. These differences were derived by dividing the mean or proportion variations by a combined SD estimate [22]. This analytical approach is not heavily influenced by sample size and is efficacious in discerning significant discrepancies, with standardized differences exceeding 0.1 being deemed significant. For UGC outcomes, the estimation of the cumulative incidence or mortality was carried out using the cumulative incidence or mortality function, with due consideration given to the competing risk of mortality or other causes. All-cause mortality was defined as any cause of mortality, and cancer-specific mortality was regarded as mortality from UGC. The Gray test was used to evaluate discrepancies between groups concerning cumulative UGC incidence and UGC mortality. All-cause mortality was assessed using the Kaplan-Meier method with a log-rank test to discern differences between groups. We used a Cox proportional hazards model to determine the respective association of endoscopy screening with each outcome. Hazard ratios (HRs) and 95% CIs were reported. All hypothesis tests were 2-sided. Statistical analyses were conducted using R software (version 3.5.1; R Foundation for Statistical Computing), considering *P* values less than .05 as indicative of statistical significance.

### Ethical Considerations

The study was approved by the ethics committee of the Cancer Hospital Chinese Academy of Medical Sciences (approval 15-070/997) and Zhejiang Cancer Hospital (approval IRB-2022‐271). All participants provided written informed consent.

## Results

In total, 153,079 participants were included in this study from 2013 to 2019. Of these, 113,916 (74.42%) of the participants were designated as low risk of UGC. The remaining 39,163 (25.68%) participants were deemed to be at high risk of UGC and were offered gastroscopy tests. Among the high-risk participants, 9627 (compliance rate 24.6%) adhered to the gastroscopy tests.

The baseline characteristics of participants in the high-risk and low-risk groups are summarized in Table 1. The mean age was 55.86 (SD 8.43) years among the entire population. The distributions of age, sex, BMI, education, marriage, and tea drinking among the high-risk group were similar to those in the low-risk group (standardized difference <0.1). In contrast to individuals in the low-risk group, participants in the high-risk group exhibited a higher likelihood of being smokers, being alcohol drinkers, being passive smokers, not performing frequent exercises, having a history of trauma and upper gastrointestinal system disease, and having family history of UGC (standardized difference >0.1).

After a median follow-up of 6.05 (IQR 3.06‐7.06) years, a total of 51 UGC cases, 2 UGC deaths, and 66 all-cause deaths were identified in the screened cohort, whereas 146 UGC cases, 40 UGC deaths, and 364 all-cause deaths occurred in the nonscreened group. There were 425 UGC cases, 138 UGC deaths, and 1528 all-cause deaths in the low-risk group. The crude UGC incidence densities were 119.2 and 98.6 per 100,000 person-years in the screened and the nonscreened cohort, respectively. Obvious reductions in both the all-cause mortality and UGC mortality were observed between the screened group (153.7 and 4.7 per 100,000 person-years) and the nonscreened group (245.3 and 27 per 100,000 person-years; Table 2).

**Figure 2.**
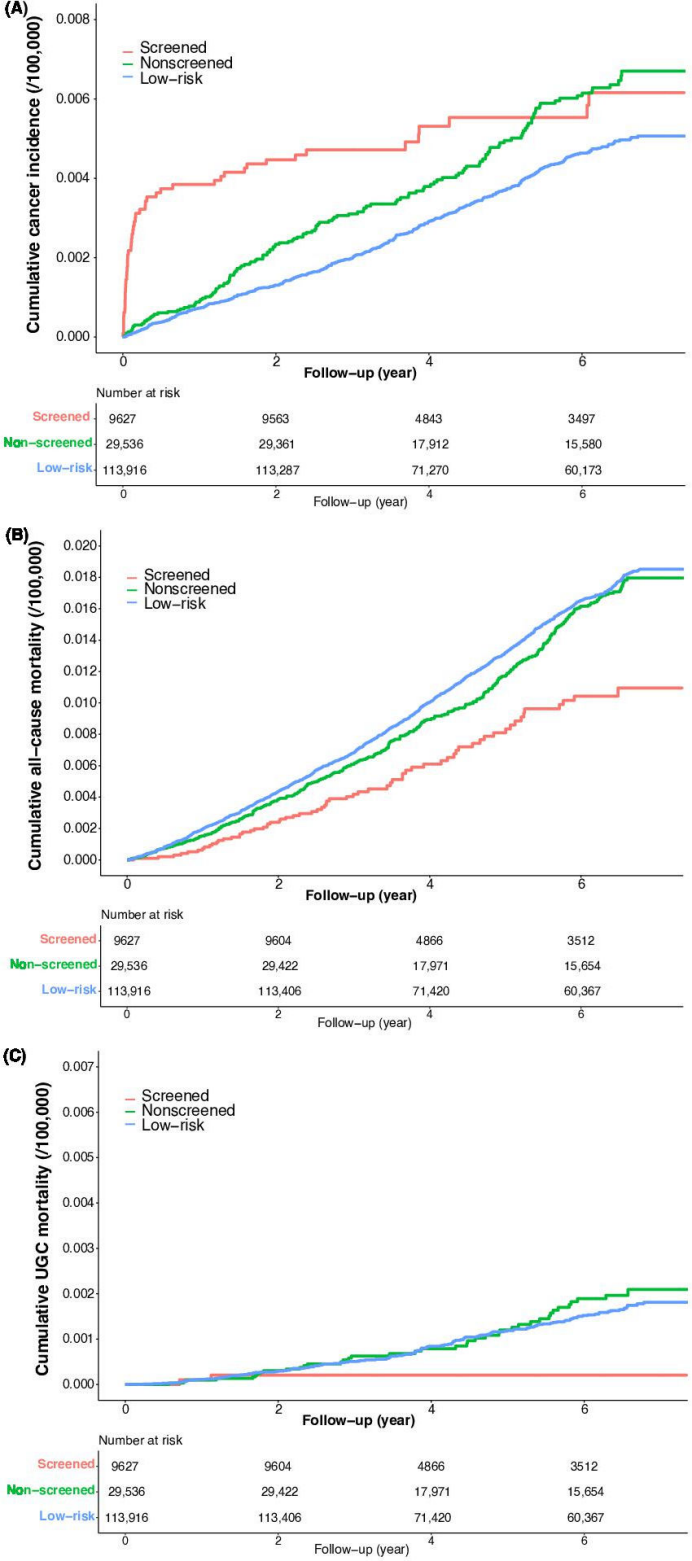
(**A**) Cumulative incidence of UGC, (**B**) all-cause mortality, and (**C**) UGC mortality. UGC: upper gastrointestinal cancer.

In Figure 2A, the screened group exhibited the highest cumulative incidence of UGC, followed by the nonscreened and low-risk groups; regarding all-cause cumulative mortality (Figure 2B), the screened group recorded the lowest rate, succeeded by the nonscreened and low-risk group; Figure 2C illustrates that cumulative UGC mortality was most pronounced in the nonscreened group, followed by the low-risk and screened group.

**Figure 3.**
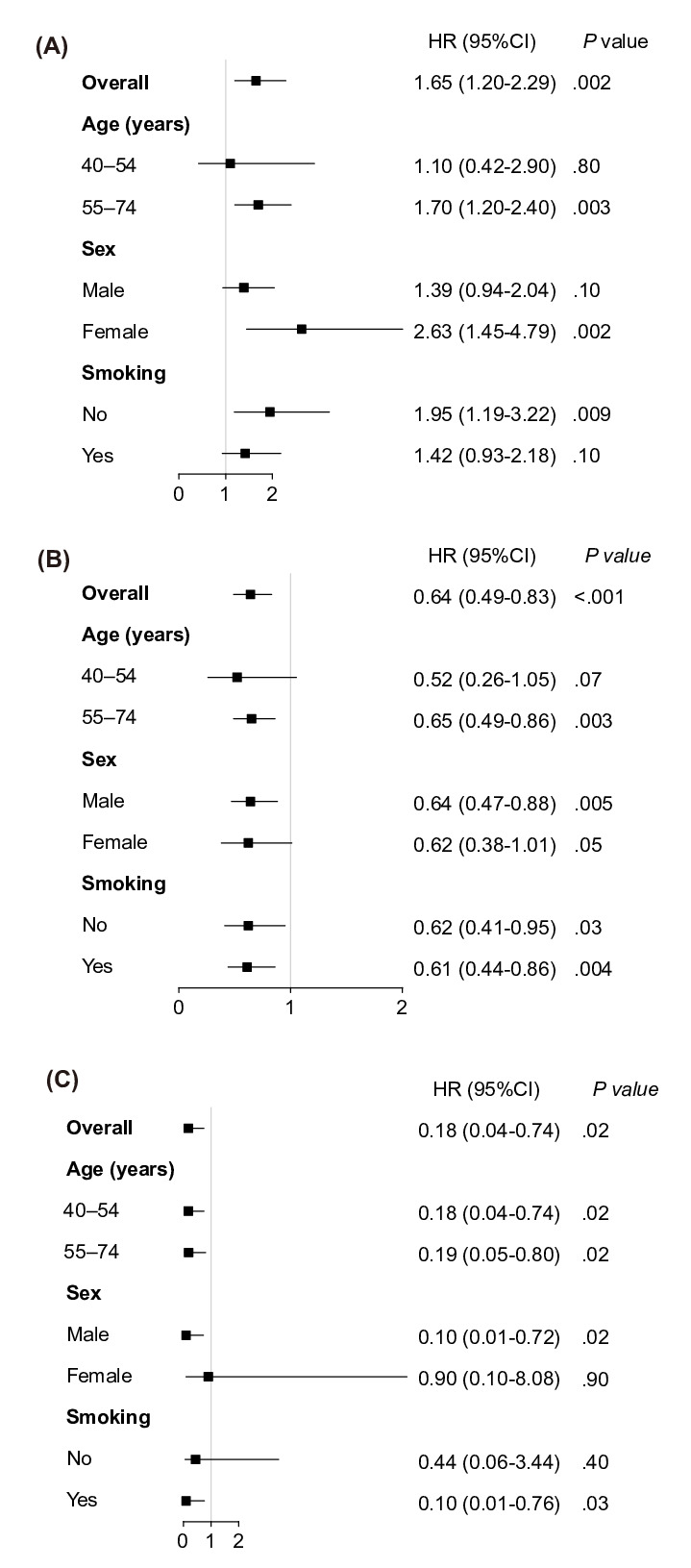
(**A**) Forest plot of the adjusted HRs for the association between an endoscopy test and incidence of upper gastrointestinal cancer, (**B**) all-cause mortality, and (**C**) upper gastrointestinal cancer mortality. Note: comparisons are between the screened group and the nonscreened group. HR: hazard ratio.

Figure 3 shows the adjusted HRs indicating the correlation between an endoscopy test and each outcome from Cox regression models. The screened group showed a significant 36% and 82% reduction in both all-cause mortality (HR 0.64, 95% CI 0.49‐0.83, *P*<.001) and UGC mortality (HR 0.18, 95% CI 0.04‐0.74, *P*=.02) compared to the nonscreened group. Reductions of 35% in all-cause mortality (HR 0.65, 95% CI 0.49‐0.86, *P*=.003) and 81% in UGC mortality (HR 0.19, 95% CI 0.05‐0.80, *P*=.02) were observed in participants aged older than 55 years in the screened group compared to the nonscreened group, and a reduction of 82% in UGC mortality (HR 0.18, 95% CI 0.04‐0.74, *P*=.02) was also observed in participants aged 40‐54 years. The reductions in all-cause mortality and UGC mortality were statistically significant in males (all-cause mortality: HR 0.64, 95% CI 0.47‐0.88, *P*=.005; UGC mortality: HR 0.10, 95% CI 0.01‐0.72, *P*=.02), but significant reductions were not observed in females (all *P* values were >.05). Compared to the nonscreened group, all-cause mortality decreased by 39% (HR 0.61, 95% CI 0.44‐0.86, *P*=.004) for smokers and 38% (HR 0.62, 95%CI 0.41‐0.95, *P*=.03) for nonsmokers in the screened group. Significant UGC mortality reductions were observed in smokers (HR 0.10, 95% CI 0.01‐0.76; *P*=.03), but not for nonsmokers (*P*=.40).

**Table 1.** Baseline characteristics of the study population from 2013 to 2019 (N=153,079).

Characteristics	Overall (n=153,079)	High-risk group				Low-risk group (n=113,916)	Standardized difference (high-risk vs low risk)
		High-risk group (n=39,163)	Screened (n=29,536)	Nonscreened (n=9627)	Standardized difference (screened vs nonscreened)		
**Age (years)**							
Mean (SD)	55.86 (8.43)	56.42 (8.08)	56.45 (8.16)	56.33 (7.81)	0.014	55.67 (8.54)	0.090
40‐54, n (%)	66,838 (43.66)	15,763 (40.25)	11,865 (40.17)	3898 (40.49)	0.007	51,075 (44.84)	0.093
55‐74, n (%)	86,241 (56.34)	23,400 (59.75)	17,671 (59.83)	5729 (59.51)	0.007	62,841 (55.16)	0.093
**Sex, n (%)**					0.027		0.008
Male	67,584 (44.15)	17,177 (43.86)	12,857 (43.53)	4320 (44.87)		50,407 (44.25)	
Female	5495 (55.85)	21,986 (56.14)	16,679 (56.47)	5307 (55.13)		63,509 (55.75)	
**BMI (kg/m** ^**2**^ **), n (%)**					0.033		0.033
<24	56,535 (57.60)	14,897 (58.80)	11,212 (59.21)	3685 (57.59)		41,638 (57.18)	
≥24	41,620 (42.40)	10,437 (41.20)	7723 (40.79)	2714 (42.41)		31,183 (42.82)	
**Education, n (%)**					0.010		0.028
No primary education	9139 (5.97)	2532 (6.47)	1928 (6.53)	604 (6.27)		6607 (5.80)	
Primary education or more	143,940 (94.03)	36,631 (93.53)	27,608 (93.47)	9023 (93.73)		107,309 (94.20)	
**Marriage, n (%)**					0.037		0.033
Unmarried	7232 (4.72)	2057 (5.25)	1610 (5.45)	447 (4.64)		5175 (4.54)	
Married	145,847 (95.28)	37,106 (94.75)	27,926 (94.55)	9180 (95.36)		108,741 (95.46)	
**Smoking, n (%)**					0.114		0.245
No	112,580 (73.54)	25,583 (65.32)	19,690 (66.66)	5893 (61.21)		86,997 (76.37)	
Yes	40,499 (26.46)	13,580 (34.68)	9846 (33.34)	3734 (38.79)		26,919 (23.63)	
**Alcohol drinking, n (%)**					0.107		0.365
No	109,242 (71.36)	23,037 (58.82)	17,757 (60.12)	5280 (54.85)		86,205 (75.67)	
Yes	43,835 (28.64)	16,126 (41.18)	11,779 (39.88)	4347 (45.15)		27,709 (24.32)	
**Passive smoking, n (%)**					0.181		0.473
No	94409 (61.67)	17496 (44.67)	13,841 (46.86)	3655 (37.97)		76,913 (67.52)	
Yes	58,668 (38.33)	21667 (55.33)	15,695 (53.14)	5972 (62.03)		37,001 (32.48)	
**Tea drinking, n (%)**					0.021		0.030
No	85,398 (55.79)	21,409 (54.67)	16,224 (54.93)	5185 (53.86)		63,989 (56.17)	
Yes	67,679 (44.21)	17,754 (45.33)	13,312 (45.07)	4442 (46.14)		49,925 (43.83)	
**Physical activity, n (%)**					0.090		0.189
No	82,587 (53.95)	23,844 (60.88)	17,665 (59.81)	6179 (64.18)		58,743 (51.57)	
Yes	70,492 (46.05)	15,319 (39.12)	11,871 (40.19)	3448 (35.82)		55,173 (48.43)	
**History of trauma, n (%)**					0.136		0.443
No	139,294 (90.99)	31,469 (80.35)	24,136 (81.72)	7333 (76.17)		107,825 (94.65)	
Yes	13,785 (9.01)	7694 (19.65)	5400 (18.28)	2294 (23.83)		6091 (5.35)	
**History of mental depression, n (%)**					0.203		0.397
No	142,098 (92.83)	3,2945 (84.12)	25,408 (86.02)	7537 (78.29)		109,153 (95.82)	
Yes	10,981 (7.17)	6218 (15.88)	4128 (13.98)	2090 (21.71)		4763 (4.18)	
**History of upper gastrointestinal system disease, n (%)**					0.252		2.165
No	115,287 (75.31)	8335 (21.28)	6995 (23.68)	1340 (13.92)		106,952 (93.89)	
Yes	37,788 (24.69)	30,827 (78.71)	22,541 (76.32)	8286 (86.07)		6961 (6.11)	
**Family history of UGC** ^**a**^ **, n (%)**					0.094		0.986
No	137,908 (90.09)	25,322 (64.66)	19,427 (65.77)	5895 (61.23)		112,586 (98.83)	
Yes	15,171 (9.91)	13,841 (35.34)	10,109 (34.23)	3732 (38.77)		1330 (1.17)	

a UGC: upper gastrointestinal cancer.

**Table 2.** Crude incidence of upper gastrointestinal cancer (UGC), all-cause mortality, and UGC mortality among all participants, high-risk group (screened and nonscreened), and low-risk group.

			Overall		High-risk group				Low-risk group	
			Number	Rate (95% CI)^a^	Screened		Nonscreened		Number	Rate (95% CI)^a^
					Number	Rate (95% CI)^a^	Number	Rate (95% CI)^a^		
**UGC** **incidence**										
	Overall		622	79.9 (73.9‐86.5)	51	119.2 (90.6‐156.9)	146	98.6 (83.9‐116.0)	425	72.4 (65.8‐79.6)
	**Age (years)**									
		40‐54	121	35.0 (29.3‐41.8)	5	29.4 (12.3‐70.7)	26	42.9 (29.2‐63.0)	90	33.5 (27.3‐41.2)
		55‐74	501	115.9 (106.2‐126.5)	46	178.3 (133.6‐238.1)	120	137.2 (114.8‐164.1)	335	105.0 (94.4‐116.9)
	**Sex**									
		Male	456	134.3 (122.6‐147.3)	34	179.7 (128.4‐251.5)	114	176.7 (147.0‐212.3)	308	120.3 (107.6‐134.5)
		Female	166	37.8 (32.5‐44.1)	17	71.3 (44.3‐114.6)	32	38.3 (27.1‐54.2)	117	35.3 (29.5‐42.3)
	**Smoking**									
		No	298	52.2 (46.6‐58.4)	22	82.0 (54.0‐124.5)	56	56.9 (43.8‐74.0)	220	49.3 (43.2‐56.3)
		Yes	324	156.7 (140.6‐174.8)	29	182.0 (126.5‐262.0)	90	181.2 (147.4‐222.8)	205	145.3 (126.7‐166.6)
**All-cause mortality**										
	Overall		1958	251.6 (240.7‐263.0)	66	153.7 (120.7‐195.6)	364	245.3 (221.3‐271.8)	1528	259.7 (247.0‐273.1)
	**Age (years)**									
		40‐54	397	114.8 (104.0‐126.6)	9	53.0 (27.6‐101.9)	64	105.6 (82.7‐135.0)	324	120.7 (108.3‐134.6)
		55‐74	1561	361.2 (343.7‐379.6)	57	221.0 (170.5‐286.5)	300	343.1 (306.4‐384.2)	1204	377.5 (356.8‐399.4)
	**Sex**									
		Male	1325	390.4 (369.9‐412.0)	47	248.4 (186.6‐330.6)	255	395.2 (349.5‐446.8)	1023	399.6 (375.9‐424.9)
		Female	633	144.3 (133.5‐156.0)	19	79.7 (50.8‐124.9)	109	130.6 (108.2‐157.5)	505	152.4 (139.7‐166.3)
	**Smoking**									
		No	1085	189.9 (178.9‐201.5)	25	93.1 (62.9‐137.8)	149	151.5 (129.0‐177.9)	911	204.2 (191.3‐217.9)
		Yes	873	422.3 (395.2‐451.3)	41	257.4 (189.5‐349.5)	215	432.9 (378.7‐494.8)	617	437.2 (404.0‐473.1)
**UGC mortality**										
	Overall		180	23.1 (20.0‐26.8)	2	4.7 (1.2‐18.6)	40	27.0 (19.8‐36.7)	138	23.5 (19.9‐27.7)
	**Age (years)**									
		40‐54	24	6.9 (4.6‐10.3)	0	—^b^	4	6.6 (2.5‐17.6)	20	7.5 (4.8‐11.6)
		55‐74	156	36.1 (30.9‐42.2)	2	7.8 (1.9‐31.0)	36	41.2 (29.7‐57.1)	118	37.0 (30.9‐44.3)
	**Sex**									
		Male	149	43.9 (37.4‐51.5)	1	5.3 (0.7‐37.5)	36	55.8 (40.2‐77.3)	112	43.8 (36.4‐52.7)
		Female	31	7.1 (5.0‐10.0)	1	4.2 (0.6‐29.8)	4	4.8 (1.8‐12.8)	26	7.8 (5.3‐11.5)
	**Smoking**									
		No	70	12.3 (9.7‐15.5)	1	3.7 (0.5‐26.4)	9	9.2 (4.8‐17.6)	60	13.4 (10.4‐17.3)
		Yes	110	53.2 (44.1‐64.1)	1	6.3 (0.9‐44.6)	31	62.4 (43.9‐88.8)	78	55.3 (44.3‐69.0)

a Rate is the number of cases per 100,000 person-years.

b Not available.

## Discussion

### Principal Findings

We conducted a multicenter, large-scale, prospective cohort study to investigate the effectiveness of sequential UGC screening in a real-world situation. Our results highlight a substantial decrease in both all-cause mortality and UGC mortality among high-risk individuals subjected to one-off endoscopic screening compared to the nonscreened group. These findings carry significant implications for clinical practice and public health, emphasizing the real benefits associated with the implementation of effective UGC screening programs.

UGC continues to represent a substantial population health challenge on a global scale. While evaluations of the program’s effectiveness have been conducted over several decades, the routine impact of the screening program remains unclear. Countries such as Japan and Korea in East Asia have also implemented nationwide population-based screening strategies to address the high burden of UGC and reduce its incidence and mortality, showing that implementing gastroscopic screening in high-risk rural areas can lower the mortality related to EC and GC [5]. The Korean National Cancer Screening Program found an overall odds ratio of GC mortality among individuals ever screened as 0.79 (95% CI 0.77‐0.81) relative to those who had never undergone screening [13,23]. On the other hand, Hamashima et al [24] revealed a standardized mortality risk ratio of 0.43 (95% CI 0.30‐0.57) for the gastroscopic screening participants in Japan. Another study illustrated decreases in cumulative mortality from UGC, with a standardized mortality rate of 0.47 (95% CI 0.25‐0.88) [25]. Despite these studies, the effectiveness of UGC screening programs remains a topic of ongoing research and discussion. The effectiveness of UGC screening in lowering mortality may be ascribed to the identification of precancerous abnormalities. The majority of cancers detected through screening were in their early stages, including precancerous lesions. These early-stage malignancies have a lower likelihood of progressing compared to advanced-stage cancers if treated promptly. Hence, the sustained decrease in UGC mortality over time aligns with the natural course of UGC, affirming the continued impact of timely intervention.

Apart from the above studies, evidence derived from large-scale, high-quality cohort studies regarding the association with all-cause mortality is sparse. One of the most compelling aspects of our results is the impact on all-cause mortality. The fact that screening significantly lowered 36% of all-cause mortality indicates that the benefits of UGC screening extend beyond merely reducing UGC-related deaths. A positive impact on overall mortality may be more conspicuous in nations with constrained medical resources and fewer diagnostic capabilities compared to countries with ample medical provisions. Individuals who participate in such screening programs experience better overall health outcomes and a lower risk of death from various causes. This suggests that the positive effects of screening are not limited to cancer-specific outcomes but can have a broader impact on an individual’s overall well-being. However, research conducted among Western populations did not consistently demonstrate a meaningful correlation [26]. The disparity could be attributed to lower UGC incidences and mortality rates in Western populations compared to East Asian populations [1]. In Europe, Areia et al [26] pointed out that the cost-effectiveness of gastroscopic screening was only evident when accompanied by colonoscopy screening in countries where the risk of GC is ≥10 per 100,000.

As expected, a greater UGC incidence density was observed in both the high-risk and screened groups, which can be attributed to increased opportunities for early diagnosis and treatment. Early detection is a key determinant of improved cancer treatment outcomes [27,28]. When UGC is diagnosed at an advanced stage, the treatment options become limited, and the prognosis tends to be less favorable. However, screening programs, as demonstrated in our study, offer a powerful tool to address this issue. One of the notable outcomes we observed was a marked increase in incidence rates within the screened group. This is a positive outcome, and it underscores the fact that screening programs effectively identify cases of UGC at earlier, more treatable stages. By increasing the opportunities for early diagnosis, screening facilitates timely medical intervention, which is known to lead to better treatment success rates. The rationale behind this increase in incidence rates within the screened group is that the screening process identifies UGC cases that might have otherwise gone undetected until they reached a more advanced and less treatable stage. When UGC is diagnosed early, patients can receive appropriate medical care, including surgical intervention, targeted therapy, or radiation treatment, all of which are associated with higher success rates in terms of disease management and patient survival. Additionally, early detection allows for a more focused and less aggressive treatment approach. This means that patients with UGC detected through screening may undergo less invasive surgical procedures, experience fewer treatment-related side effects, and have a higher likelihood of achieving complete remission. As a result, the quality of life for these individuals can be significantly improved.

We also highlight variations in the effectiveness of screening among different age groups and genders. Notably, the benefits of screening appear more pronounced in older participants, particularly concerning UGC mortality. This may be linked to age-related differences in cancer progression and treatment response. It has been reported that advanced age is associated with an increased risk of developing UGC [29]. The UGC incidence, all-cause mortality, and UGC mortality in the age group of 55‐74 years are all remarkably higher than those in the age group of 40‐54 years, consistent with findings from other studies [30,31]. In addition, our study showed that older adult individuals showed markedly reduced risks of all-cause mortality (HR 0.65, 95% CI 0.49‐0.86) and UGC mortality (HR 0.19, 95% CI 0.05‐0.80) after the endoscopic screening in contrast to the younger group. Furthermore, we observed a more pronounced effect of screening in males, with significant reductions in both all-cause mortality and UGC mortality, whereas the effect was less significant in females. This could reflect gender-specific disparities in UGC incidence and survival. These differences may be associated with unfavorable lifestyle habits in males, such as smoking, alcohol consumption, and irregular dietary patterns [32]. Moreover, we observed similar reductions in all-cause mortality for smokers (0.61, 95% CI 0.44‐0.86) and nonsmokers (0.62, 95% CI 0.41‐0.95) after endoscopic screening, which means the screening initiative contributes to overall health outcomes, irrespective of smoking status. While the study reveals significant reductions in UGC mortality among smokers (HR 0.10, 95% CI 0.01‐0.76), the absence of a similar effect in nonsmokers raises intriguing questions. The explanation for this may lie in several factors, including distinct etiological pathways of UGC in smokers and nonsmokers, or differences in health awareness among these 2 populations. Further exploration of these nuances could enhance our understanding of the intricate interplay among risk exposures, screening effectiveness, and mortality outcomes.

### Limitations

Our study has several limitations. First, the follow-up duration of 6.05 years might be insufficient for capturing all death cases, potentially leading to an underestimation of the long-term impact of the screening program. Ideally, a more extended follow-up period would afford a comprehensive understanding of sustained benefits and potential late-emerging effects. Future research endeavors should consider prolonging follow-up periods to fully evaluate the program’s long-term effectiveness. Second, one must consider that the findings might not be universally representative across the entire general population of China. However, the study does offer a valuable scientific foundation for areas exhibiting equivalent socioeconomic profiles. Third, the screening compliance rate of 24.6% among high-risk individuals raises concerns about the generalizability of the findings to the entire high-risk individuals. The relatively low uptake rate may give rise to selection bias, influencing observed outcomes. However, it marginally surpassed the figure reported for UGC screening in Henan Province (18.4%) and Liaoning Province (22.3%), China [10,31,32]. Future research could explore strategies to enhance participation, such as community engagement programs, targeted awareness campaigns, or initiatives aimed at overcoming potential barriers to screening. These measures would ensure a more representative study population and enhance the generalizability of the findings. Future randomized controlled trials may be warranted to further validate the efficacy of risk-adapted sequential screening strategies.

### Conclusions

In conclusion, our study suggests the significance of UGC screening in reducing both all-cause mortality and UGC mortality, particularly among high-risk individuals. Screening offers the opportunity for early UGC detection, enhancing treatment success rates and alleviating the disease burden for patients. However, screening effectiveness may vary by age and gender, necessitating consideration when implementing screening programs. Our research provides robust support for further exploration and implementation of UGC screening, with the potential to alleviate the disease burden of UGC.

## Supplementary material

10.2196/62864 Multimedia Appendix 1 The flow of risk estimation process [21].

## Abbreviations

CanSPUC: Cancer Screening Program in Urban China
EC: esophageal cancer
GC: gastric cancer
HR: hazard ratio
UGC: upper gastrointestinal cancer
