# Correction: Effectiveness of Risk-Adapted Upper Gastrointestinal Cancer Screening in China: Prospective Cohort Study

**DOI:** 10.2196/67642

**Published:** 2024-11-06

**Authors:** Youqing Wang, Juan Zhu, Huizhang Li, Le Wang, Chen Zhu, Xue Li, Shi Wang, Lingbin Du

**Affiliations:** 1 Department of Cancer Prevention, Zhejiang Cancer Hospital, Hangzhou Institute of Medicine (HIM), Chinese Academy of Sciences, Hangzhou, China; 2 Department of Endoscopy, Zhejiang Cancer Hospital, Hangzhou Institute of Medicine (HIM), Chinese Academy of Sciences, Hangzhou, China

In “Effectiveness of Risk-Adapted Upper Gastrointestinal Cancer Screening in China: Prospective Cohort Study” (JMIR Public Health Surveill 2024;10:e62864) the authors noted two errors.

Affiliation 1 originally appeared as:

^1^Department of Cancer Prevention, Hangzhou Institute of Medicine (HIM), Chinese Academy of Sciences, Zhejiang Cancer Hospital, Hangzhou, China

This has been revised to:

^1^Department of Cancer Prevention, Zhejiang Cancer Hospital, Hangzhou Institute of Medicine (HIM), Chinese Academy of Sciences, Hangzhou, China

Please note that the Corresponding Author’s address information has also been updated to reflect the above change.

Additionally, Affiliation 2 was previously listed as:

^2^Department of Endoscopy, Hangzhou Institute of Medicine (HIM), Chinese Academy of Sciences, Zhejiang Cancer Hospital, Hangzhou, China

This has been revised to:

^2^Department of Endoscopy, Zhejiang Cancer Hospital, Hangzhou Institute of Medicine (HIM), Chinese Academy of Sciences, Hangzhou, China

The correction will appear in the online version of the paper on the JMIR Publications website on November 6, 2024, together with the publication of this correction notice. Because this was made after submission to PubMed, PubMed Central, and other full-text repositories, the corrected article has also been resubmitted to those repositories.

