# Supplementary material for: Effectiveness of Risk-Adapted Upper Gastrointestinal Cancer Screening in China: Prospective Cohort Study
Source: JMIR Public Health Surveill. 2024 Oct 10;10:e62864. doi: 10.2196/62864 (PMC11486449; doi:10.2196/62864)
Supplement: Multimedia Appendix 1 [file publichealth-v10-e62864-s001.docx]

We adopted the sex-specific risk score systems derived from the Harvard Cancer Risk Index to evaluate the risk of UGC [21]. Each risk factor was assigned a score by an expert panel based on the magnitude of its association with UGC. Cumulative risk scores were calculated and divided by the average risk score in the general population, yielding final individual relative risks. The flow of risk estimation process is summarized in the Figure below, the steps are:

1. We estimate risk points for each risk factor based on a simple, usually dichotomous, response.

2. Sum these for the individual.

3. Divide these by the population average.

4. Multiply the level of risk by the average rate to estimate the risk of cancer diagnosis over the next 10 years.

We adopted a group consensus process to identify the genetic, environmental, nutritional, and lifestyle factors, as well as major illnesses that are established or likely causes of UGC. In a manner similar to the International Agency for Research on Cancer (IARC) criteria, the causes were classified as definite, probable, and possible. This allowed us to separate the strength of evidence from the magnitude of the association between a risk factor and cancer. We use only the definite and probable causes in our subsequent risk index. For each exposure, a relative risk was identified as the most likely descriptor of the association in humans. To avoid protracted debate regarding the precise magnitude of the association we used five categories of relative risk (none RR = 0.9 to 1.19; weak RR = 1.2 to <1.5; moderate RR = 1.5 to <3.0; strong RR = 3.0 to <7.0; very strong RR = 7 or more). We next developed the cancer risk point scale to translate relative risks into risk points. The highest allowed risk point was 50 for a relative risk of 7 or more. Average population risk was calculated to be 16 points. To compare risk of cancer for an individual against average population, we divided the risk points for the particular individual by the population average.
